# Supplementary material for: Mid-Regional Pro-Adrenomedullin as a Prognostic Factor for Severe COVID-19 ARDS
Source: Antibiotics (Basel). 2022 Aug 29;11(9):1166. doi: 10.3390/antibiotics11091166 (PMC9495198; doi:10.3390/antibiotics11091166)
Supplement: Supplementary file 1 [file antibiotics-11-01166-s001.zip › antibiotics-1868794-supplementary.pdf]

Supplementary material

Mid-regional Pro-adrenomedullin as a prognostic factor for severe COVID-19 ARDS

Etienne de Montmollin, Katell Peoc'h, Mehdi Marzouk, Stéphane Ruckly, Paul-Henri Wicky, Juliette Patrier, Pierre Jacquet, Romain Sonneville, Lila Bouadma, Jean-François Timsit

Figure S1. Study flowchart.

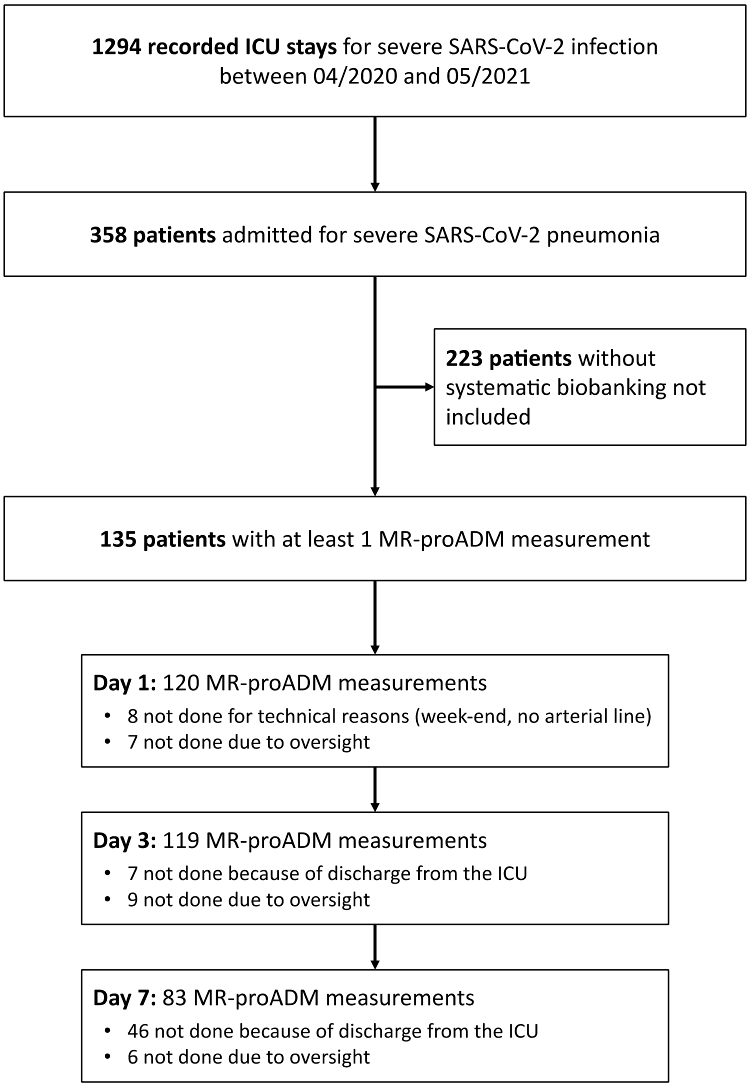

Table S1. Landmark analysis of the association of day-3 and day-7 MR-proADM with day-60 mortality, according to day-1 MR-proADM.

	HR	95% CI	p
**Landmark at day 1 (n = 135)**			
Day-1 MR-proADM	1.16	(1.07–1.26)	<.001
**Landmark at day 3 (n = 135)**			
Day-1 MR-proADM	1.09	(0.98–1.21)	0.102
MR-proADM delta between day 3 and day 1	1.20	(1.01–1.43)	0.039
**Landmark at day 7 (n = 128)**			
Day-1 MR-proADM	1.21	(1.10–1.33)	<.001
MR-proADM delta between day 7 and day 1	1.26	(0.90–1.76)	0.163

Missing data imputed by multiple imputation. Hazard ratios computed per one unit. Abbreviations: HR, Hazard Ratio; CI, Confidence Interval; MR-proADM, Mid-regional proadrenomedullin.

Figure S2. Survival curves according to a cut-point of 1 nmol/L of MR-proADM, on days 3 and 7.

A. Survival curve according to Day-3 MR-proADM

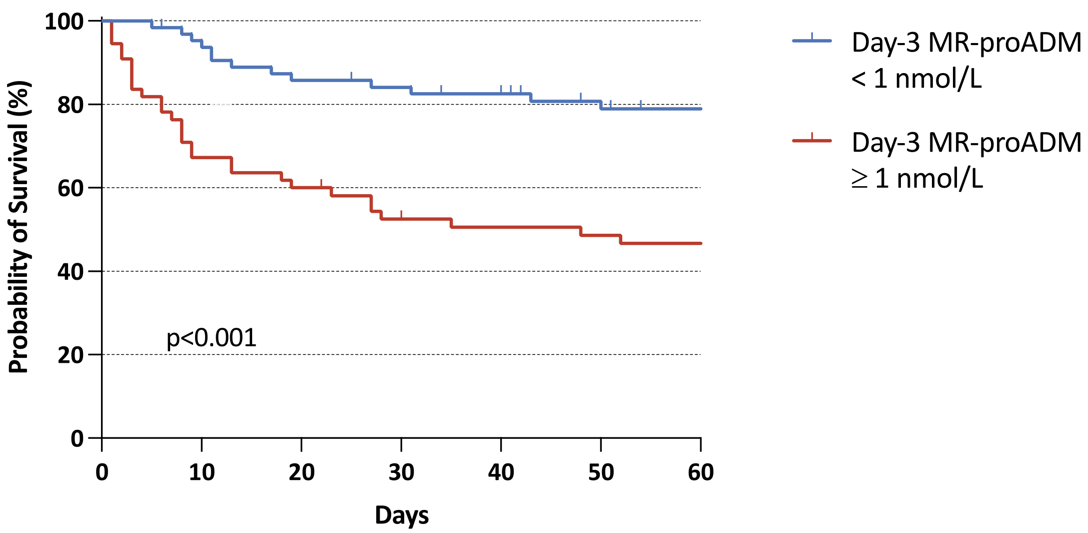

B. Survival curve according to Day-7 MR-proADM

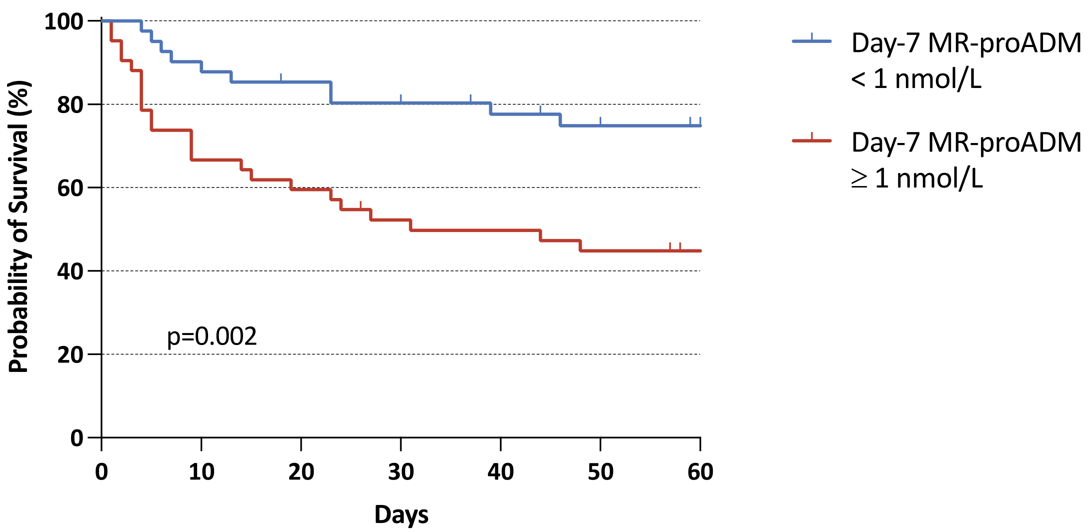

Abbreviations: MR-proADM, Mid-regional proadrenomedullin

p computed by the log-rank test.
